# Prospective association between depressive symptoms and stroke risk among middle-aged and older Chinese

**DOI:** 10.1186/s12888-021-03492-9

**Published:** 2021-10-27

**Authors:** Yimin Cui, Chunsu Zhu, Zhiwei Lian, Xueyan Han, Qian Xiang, Zhenming Liu, Ying Zhou

**Affiliations:** 1 Department of Pharmacy, Peking University First Hospital, No. 6, Dahongluochang Street, Xicheng District, Beijing, 100034 China; 2 School of Pharmaceutical Sciences, Peking University, Beijing, China; 3 Fujian Medical University Cancer Hospital, Fujian Cancer Hospital, Fuzhou, China; 4 Department of Medical Statistics, Peking University First Hospital, Beijing, China

**Keywords:** Depressive symptoms, Epidemiology, Prospective analysis, Stroke, Middle-aged, Older adults

## Abstract

**Background:**

This study aimed to assess the association between baseline symptoms and changes in depressive symptoms and stroke incidents.

**Methods:**

We used data from the Chinese Health and Retirement Longitudinal Study conducted in 2013, 2015, and 2018. In total, 10,100 individuals aged ≥45 years and without a history of stroke in 2013 were included. Depressive symptoms were measured using the 10-item version of the Center for Epidemiological Studied Depression scale (elevated depressive symptoms cutoff ≥10). Changes of depressive symptoms were assessed by two successive surveys (stable low/no, recent onset, recently remitted, and stable high depressive symptoms). We assessed whether baseline depressive symptoms and changes of them were associated with stroke incidents reported through 2018. Logistic regression analyses adjusted for age, gender, education, marital status and other potential confounders were performed.

**Results:**

For the analysis of baseline depressive symptoms and stroke (*n* = 10,100), 545 (5.4%) reported stroke incidents in the following 5-year period. Individuals with elevated depressive symptoms in 2013 experienced a markedly higher stroke risk (odds ratio (OR) = 1.53, 95% confidence interval (CI) = 1.28–1.84) compared with those without elevated depressive symptoms. In the analysis of changes in depressive symptoms (*n* = 8491, 430 (5.1%) stroke events), participants with stable high (OR = 2.01, 95% CI = 1.58–2.56) and recent-onset (OR = 1.39, 95% CI = 1.04–1.85) depressive symptoms presented higher stroke risk compared to those with stable low/no depressive symptoms, while recently remitted symptoms (OR = 1.12, 95% CI = 0.80–1.57) were not associated with stroke risk.

**Conclusions:**

In conclusion, stable high and newly started depressive symptoms were associated with increased stroke risk, whereas the improvement of depressive symptoms was not related to increase in stroke risk, suggesting that stroke risk may be decreased by effective management of depressive symptoms.

**Supplementary Information:**

The online version contains supplementary material available at 10.1186/s12888-021-03492-9.

## Background

Stroke is the second major cause of death and a leading cause of disability worldwide, causing nearly 5% of all disability-adjusted life years and 10% of all deaths [1, 2]. The overall burden of stroke is substantial and increasing globally, although the age-standardized stroke mortality rate has decreased worldwide in recent decades [3]. The mortality rate is relatively high in Asia, contributing to a more serious stroke burden than that in Europe or the US [4]. In China, 13 million people have suffered a stroke, and it is the primary cause of death [5]. Depression is a common psychiatric disorder, affecting almost 3500 million people worldwide [6]. In China, 6.9% of adults have reported depression [7], and nearly 40% of the population aged more than 60 has suffered from depressive symptoms [8]. Depression may increase stroke risk through several potential pathways, including dysregulation of the immune system and induction of inflammation [9], unhealthy behaviors [10], decreased medication adherence [11] and the development of hypertension and diabetes [12, 13].

Previous prospective and cross-sectional studies have assessed the relationship between depression and stroke risk; they were mainly conducted in developed countries and reported a positive association between depression and risk of stroke [14–16]. However, most of that research have only assessed the baseline state of depressive symptoms [17–21], and have not accounted for the fact that depression is a time-varying variable that may be relapsing or remitting. Most people with depression remain untreated and might suffer from fluctuating depressive symptom levels [22]. Thus, measuring depressive symptoms as a dynamic variable may provide more precise estimates of the relationship between depressive symptoms and stroke risk. However, few studies have examined the effects of changes in depressive symptoms on stroke risk, with only three studies conducted in the US or Mexico, and they produced mixed results [23–27]. The results from these countries may not be generalized to China because there are several differences in cardiovascular risk factors and outcomes. For example, stroke is the leading cause of death in China, but not in Western countries [5].

To the best of our knowledge, a few studies have been conducted in China and have found a marginally significant correlation between depressive symptoms and stroke risk in the general population [21], and no study has investigated the associations between changes in depressive symptoms and stroke risk in Chinese people. Therefore, the objectives of this study were twofold. First, we assessed the effect of baseline depressive symptoms on stroke risk among Chinese adults aged ≥45. Second, we examined the association between changes in depressive symptoms and stroke risk in this population. These findings provide insights into their relationship and may help to better understand whether successful intervention and treatment of depressive symptoms is likely to lower stroke risk.

We hypothesized that baseline elevated depressive symptoms would be associated with a greater stroke risk. We also expected that recent-onset and stable high depressive symptoms would be significantly associated with stroke risk compared to stable low/no depressive symptoms. Furthermore, we expected that remission of symptoms would be insignificantly associated with stroke.

## Methods

### Study population

The study data were derived from the Chinese Health and Retirement Longitudinal Study (CHARLS): a nationwide, community-based cohort study of population aged ≥45 for open access. Based on the American Health and Retirement Study (HRS) design, the CHARLS surveyed 150 county-level units in 28 provinces in mainland China every two years. Face-to-face, computer-assisted personal interviews (CAPI) were conducted in interviewees’ homes by trained interviewers. To ensure the representativeness of the sample, multi-level stratified sampling was implemented. Three levels of sampling frames were included (county/city, village/neighborhood committee and households). With probability proportional to size (PPS) method, 150 counties or cities were selected from 30 provinces in mainland China. Then three villages or neighborhood committees were selected with PPS from each county or city. Within each village or neighborhood committee, 80 households were randomly selected based on a specialized Geographic Information System program. Then within each household, one adult aged 45 years or above as well as his/her spouse were randomly selected to be included in the survey. A detailed description of the project and its procedures are provided elsewhere [28].

**Fig. 1:**
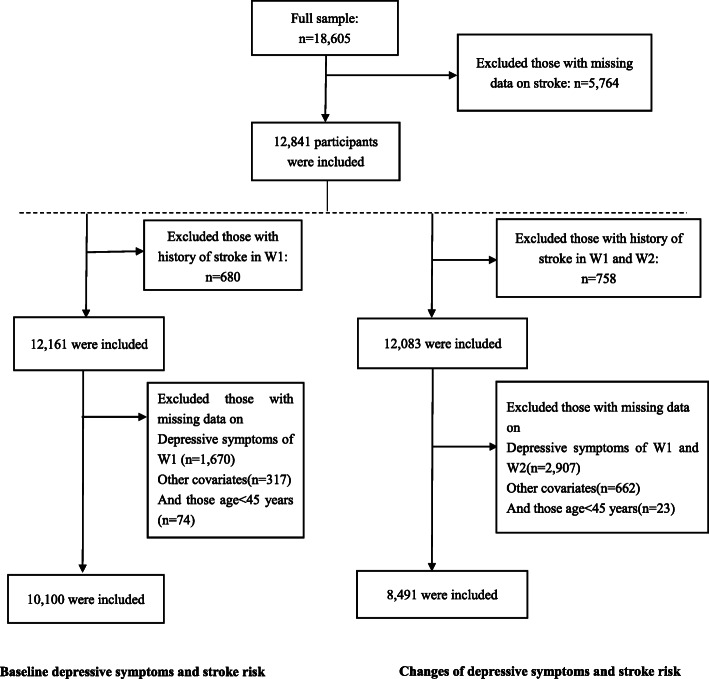
Flowchart of study participants’ selection

W1: Wave 1, the survey of the China Health and Retirement Longitudinal Study carried out in 2013; W2: Wave 2, the interview conducted in 2015.

In the current analysis, we included data from the CHARLS conducted in 2013 (Wave 1, W1), 2015 (Wave 2, W2), and 2018 (Wave 3, W3). A total of 18,605 individuals were interviewed in 2013. We excluded participants for the following reasons: under 45 years, history of stroke, missing data on stroke, primary exposure, and other covariates. The study participants’ selection process is shown in Fig. 1. For the baseline depressive symptoms and stroke risk analysis, 10,100 individuals were included, while, for the changes in depressive symptoms analysis, 8491 individuals were included. The baseline demographic characteristics among all the participants and those included in our study were largely comparable. This study followed the Strengthening the Reporting of Observational Studies in Epidemiology (STROBE) guideline (Additional file 2: Appendix 1).

### Stroke outcomes

A stroke event was defined as the first non-fatal or fatal stroke, based on self- or proxy-reported doctor diagnosis (“Have you been diagnosed with stroke by a doctor?”). In cases where the subject could not be interviewed directly, the interview was conducted through a proxy (such as a spouse or another relative).

### Primary exposure

Depressive symptoms were measured using the 10-item version of the Center for Epidemiological Studied Depression scale (CESD-10), querying the positive and negative emotions of the participants in the prior week. The CESD-10 consists of eight negative items and two positive items. Each item was coded as 0, 1, 2, and 3 scores, and positive items were reverse coded. The total depressive symptoms score was calculated by summing scores across all items for a maximum score of 30. For each exposure wave, a total score of at least 10 was classified as elevated depressive symptoms. Previous studies have shown that the CESD-10 has high reliability and validity for older Chinese people [29, 30].

Changes in depressive symptoms were defined based on W1 and W2 interviews, and we categorized depressive symptoms into four exposure patterns (1): stable high: elevated depressive symptoms at both W1 and W2 (2); recent onset: elevated depressive symptoms at W2, but not at W1 (3); recently remitted: elevated depressive symptoms at W1, but not at W2 (4) stable low/no: no elevated depressive symptoms at both W1 and W2 [23–25].

### Covariates

We considered several potential confounders in this study: demographic, behavior-related, and biological variables at baseline. The demographic variables included age, gender, education (less than high school, high school education and above), marital status (married and single—the latter including those who were widowed, separated, divorced, or never married), and place of residence (urban or rural). Behavior-related variables included smoking status (never smoker, past smoker, and current smoker), and drinking frequency (never, rarely, and often). Biological variables included body mass index (BMI), hypertension (yes/no), diabetes (yes/no), and heart diseases (yes/no). Hypertension status was determined based on the history of hypertension, intake of antihypertensive drugs, and measurement of blood pressure (systolic blood pressure ≥ 140 mmHg or diastolic blood pressure ≥ 90 mmHg). Diabetes diagnosis was based on self-reported medical history and blood measurements provided by the staff from the Chinese Center for Disease Control and Prevention (CDC). Heart diseases were defined by a self-reported doctor diagnosis.

### Statistical analysis

Descriptive statistics of baseline characteristics were presented according to the four depressive symptom categories, continuous variables were presented as mean and standard deviation (SD), and categorical variables were shown as absolute and relative frequencies. Analysis of variance was used to compare continuous data of different depressive symptom patterns, and the chi-square test was used to compare categorical variables. Missing value analyses were conducted to identify the mechanisms of missing variables, and based on the results, we hypothesized that the data used in our study were missing at random [31]. In order to estimate the association between changes in depressive symptoms and stroke incidents, we fitted three binary logistic regression models, with stable low/no as the referent group. Model 1 was unadjusted in order to calculate the crude odds ratio (OR) of stroke risk. In Model 2, baseline demographic characteristics (including age, gender, education, marital status, and place of residence) were added. In Model 3, potential stroke risk factors including smoking status, drinking frequency, BMI, hypertension, diabetes, and heart diseases were further adjusted [17, 32, 33]. We retained the variables, even if they were not statistically significant in changing the association between changes in depressive symptoms and stroke risk (*p* ≥ .05), to see if the changes in depressive symptoms were independent of these variables. To estimate the association between baseline depressive symptoms and stroke risk, series of similar logistic regressions were conducted with baseline depressive symptoms as primary exposure. In addition to these analyses, we examined the association between continuous CESD-10 score to determine the risk of stroke associated with 1-unit increase in CESD-10. Stratification analyses by baseline age, gender, and place of residence were also conducted to investigate the association in different subgroups. Then, interaction analyses between depressive symptoms and the aforementioned variables were further carried out. Finally, to test the robustness of our findings, we performed sensitivity analyses by defining elevated depressive symptoms with an alternative cutoff (CESD-10 ≥ 12), which was reported to have a higher sensitivity to identify clinically depressive symptoms in older Chinses, and then repeating the logistic regression analyses [34]. Two-sided *P*-values <.05 were considered statistically significant. All analyses were conducted using the Statistical Package for the Social Sciences (SPSS), version 21.

## Results

### Baseline depressive symptoms and stroke risk

The analysis that estimated the effect of baseline depressive symptoms on stroke risk included 10,100 individuals; 3099 (30.7%) had elevated depressive symptoms at baseline (W1), and 545 (5.4%) had reported stroke events during the following 5-year period. The mean age of this sample was 59.6, and 5425 (53.7%) were women (Supplementary Table I). Participants with elevated depressive symptoms were more likely to be women, with a low education level, single, living in rural areas, and with heart disease.

Compared to those with no elevated depressive symptoms at baseline, participants reporting elevated depressive symptoms had a markedly increased risk of stroke incidents in Model 1 (OR = 1.52, 95% CI = 1.27–1.81). The risk of stroke for 1-unit increase in the CESD-10 score (continuous variable) was 1.04 (95% CI = 1.02–1.05) (see Supplementary Table II). The results were similar after adjusting for demographic characteristics in Model 2. In Model 3, after fully adjusting for age, sex, education, marital status, place of residence, smoking status, drinking frequency, BMI, hypertension, diabetes, and heart disease, individuals with elevated depressive symptoms had a higher stroke risk (OR = 1.53, 95% CI = 1.28–1.84) than those with no elevated depressive symptoms. The corresponding fully adjusted OR for 1-unit increase on the CESD-10 score (continuous variable) was 1.04 (95% CI = 1.02–1.05) (Supplementary Table II). There were no interactions by baseline age (*P* = .95), gender (*P* = .41) and place of residence (*P* = .19) in the fully adjusted model (Supplementary Table III).

### Changes in depressive symptoms and stroke risk

A total of 8491 participants were included in the analysis of changes in depressive symptoms. There were 2612 (30.8%) and 2852 (33.6%) participants who had a depression rating score of 10 or greater at W1 and W2, respectively, indicative of elevated depressive symptoms. The mean age was 59.4 years, 4688 (55.2%) of them were women, and the majority were married (Table 1). Participants with stable low/no depressive symptoms were the most commonly reported depressive symptom pattern 4646 (54.7%), followed by the stable high group, 1619 (19.1%), recent onset group, 1234 (14.5%), and recently remitted group, 992 (11.7%). Most of the patients with stable high depressive symptoms were women, living in rural areas, with low education, single, and with heart disease (Table 1).

**Table 1:** Participant characteristics at baseline, 2013, stratified by changes in depressive symptom groups, *n* = 8491

Characteristics	Overall, n = 8491	Changes in depressive symptoms				***P*** value
		Stable low ***n*** = 4646	Recent onset ***n*** = 1234	Recently remit ***n*** = 992	Stable high ***n*** = 1619	
Age, Mean (SD), y	59.4 (8.3)	59.3 (8.4)	59.4 (8.2)	59.5 (8.5)	59.6 (8.0)	.546
Female, n (%)	4688 (55.2)	2228 (48.0)	744 (60.3)	584 (58.9)	1132 (69.9)	<.001
Married, n (%)	7630 (89.9)	4259 (91.7)	1106 (89.6)	885 (89.2)	1380 (85.2)	<.001
Above high school education, n (%)	976 (11.5)	634 (13.6)	101 (8.2)	108 (10.9)	133 (8.2)	<.001
Live in rural, n (%)	7835 (92.3)	4198 (90.4)	1167 (94.6)	936 (94.4)	1534 (94.7)	<.001
Smoking status, n (%)						<.001
Never smoker	5225 (61.5)	2681 (57.7)	776 (62.9)	635 (64.0)	1133 (70.0)	
Current smoker	2727 (32.1)	1636 (35.2)	373 (30.2)	310 (31.3)	408 (25.2)	
Past smoker	539 (6.3)	329 (7.1)	85 (6.9)	47 (4.7)	78 (4.8)	
Drinking status, n (%)						
Never	5512 (64.9)	2824 (60.8)	835 (67.7)	687 (69.3)	1166 (72.0)	<.001
Rarely	702 (8.3)	409 (8.8)	89 (7.2)	75 (7.6)	129 (8.0)	
Often	2277 (26.8)	1413 (30.4)	310 (25.1)	230 (23.2)	324 (20.0)	
BMI, kg/m^2^, Mean (SD)	24.0 (4.8)	24.2 (4.3)	24.1 (7.3)	24.0 (4.5)	23.6 (3.8)	.001
Hypertension, n (%)	3827 (45.1)	2073 (44.6)	572 (46.4)	435 (43.9)	747 (46.1)	.467
Diabetes, n (%)	1000 (11.8)	526 (11.3)	156 (12.6)	124 (12.5)	194 (12.0)	.497
Heart disease, n (%)	1073 (12.6)	516 (11.1)	132 (10.7)	148 (14.9)	277 (17.1)	<.001

Abbreviations: SD = standard deviation. Analysis of variance was used for continuous variable, chi-square test for categorical variables

Of all subjects with no history of stroke before W3, 430 (5.1%) had reported stroke incidents during the following 3-year period. The results of the binary logistic regression analysis are shown in Table 2. In Model 1, compared to those with stable low/no depressive symptoms, those who had stable high depressive symptoms had higher probability of stroke incidents (OR = 2.01, 95% CI = 1.59–2.53), and recently manifested depressive symptoms were also associated with increased stroke incidents (OR = 1.40, 95% CI = 1.05–1.85). No significant associations were detected among the recently remitted group (OR = 1.12, 95% CI = 0.80–1.56). In Model 2, after additional adjustment for baseline age, gender, education, marital status, and place of residence, the ORs were slightly altered, but the statistical significances remained similar. In Model 3, after fully adjusting for age, gender, education, marital status, place of residence, smoking status, drinking frequency, BMI, hypertension, diabetes, and heart disease, patients with stable high and recent-onset depressive symptoms had, respectively, 101% (OR = 2.01, 95% CI = 1.58–2.56) and 39% (OR = 1.39, 95% CI = 1.04–1.85) higher stroke risk than did patients with stable low/no depressive symptoms. No significant associations were found among patients with remitted depressive symptoms (*p* > .05). Subgroup analyses and interaction analyses results are presented in Supplementary Table IV. The *P*-values for interactions assessing possible differences in effect by baseline age (.22), gender (.73), and place of residence (.10) were not statistically significant.

**Table 2:** Odds Ratios and 95%CI for associations between changes in depressive symptoms and stroke incidents

	Model 1		Model 2		Model 3	
Depressive Symptom Patterns	OR (95%CI)	P value	OR (95%CI)	P value	OR (95%CI)	P value
Stable low	Ref		Ref		Ref	
Recent onset	1.40 (1.05,1.85)	.021	1.42 (1.07,1.89)	.016	1.39 (1.04,1.85)	.026
Recently remitted	1.12 (0.80,1.56)	.502	1.13 (0.81,1.67)	.487	1.12 (0.80,1.57)	.512
Stable high	2.01 (1.59,2.53)	<.001	2.03 (1.60,2.58)	<.001	2.01 (1.58,2.56)	<.001

Abbreviations; OR = odds ratio, CI = confidence interval

The stable low/no group was used as the reference. The model 1 was unadjusted. The model 2 was adjusted by baseline demographic variables including age, gender, education, marital status, place of residence. The model 3 was further adjusted by smoking status, drinking frequency, body mass index, hypertension, diabetes and heart disease

The stable low/no group was used as the reference. The Model 1 was unadjusted. The Model 2 was adjusted by baseline demographic variables including age, gender, education, marital status, place of residence. The Model 3 was further adjusted by smoking status, drinking frequency, BMI, hypertension, diabetes, and heart disease.

### Sensitivity analyses

In sensitivity analyses (Supplementary Table V), where elevated depressive symptoms were defined by a higher cutoff (CESD-10 ≥ 12), participants reporting elevated depressive symptoms at baseline had a 1.36-fold higher risk of stroke compared to those with no elevated symptoms in the fully adjusted model. Stable high (OR = 2.03, 95% CI = 1.56–2.64) and recent onset (OR = 1.53, 95% CI = 1.16–2.03) depressive symptoms were statistically associated with increased stroke risks in comparison to stable low/no group. Yet no significant associations were detected between recently remitted of depressive symptoms and stroke risk (*p* > .05).

## Discussion

In this nationally representative cohort study of middle-aged and older Chinese populations, we found evidence of increased stroke risk for participants reporting elevated depressive symptoms at baseline. Participants with stable high depressive symptoms over two successive assessments had 101% more stroke incidents reported during the next 3-year period, compared with participants with stable low/no depressive symptoms, after adjustment for potential confounders. Compared to those with stable low/no depressive symptoms, subjects with recent onset of depressive symptoms had a 39% increase in stroke risk. Consistent with our hypothesis, the recent remittance of depressive symptoms had no significant association with increased incidence of stroke. There was no evidence of interaction effects of gender, age, or place of residence.

Our findings regarding the association between baseline depressive symptoms and stroke risk in the CHARLS were consistent with several previous studies [35–37]. Two recent meta-analyses, reporting an adjusted hazard ratio of 1.45 (95% CI = 1.29–1.63) and pooled relative risk of 1.34 (95% CI = 1.17–1.54) also supported our results [38, 39]. Moreover, the results were largely consistent with our hypothesis that individuals with new symptom onset and stable high depressive symptoms across two consecutive biennial assessments have increased stroke incidents during the following 3-year period, compared with individuals with stable low/no depressive symptoms. This was in line with previous results from the Cardiovascular Health Study (CHS) in the US [24], which found that individuals with persistently high and recent-onset depressive symptoms across annual assessments had 65 and 44% increased hazards of stroke incidence in the following year, respectively. The Mexican Health and Aging Study (MHAS) examined the effect of baseline short-term changes in depressive symptoms on stroke incidents reported over a 12-year follow-up, and found that participants with recent-onset and stable high depressive symptoms presented 38 and 42% higher risk of stroke, respectively, than those with stable low/no depressive symptoms, whereas recently remitted symptoms were not associated with stroke risk [27]. The HRS is a sister study of the CHARLS conducted in the US. The results also showed that stable high depressive symptoms were related to double stroke risk compared with the stable low/no depressive symptoms group. However, the results of the HRS were inconsistent with our findings to some extent, because they suggested that recently remitted depressive symptoms—rather than the recent onset of depressive symptoms—were associated with increased stroke incidents [26]. The possible cause of the differences may be related to the relatively short follow-up waves in the CHARLS, which prevented us from using the same statistical techniques as prior studies. However, two prior studies might support our results. Moise et al. prospectively assessed the association between elevated depressive symptoms and stroke among 22,666 black and white middle-aged and older participants, and found that proximal rather than distal depressive symptoms were associated with stroke incidents [40]. Pan et al. estimated the effects of a prior or current diagnosis of depression on stroke risk and found that patients with a prior history of depressive symptoms had an insignificantly elevated risk of stroke, while those currently diagnosed with depression had an increased stroke risk [41].

The mechanism linking depressive symptoms and stroke remains unclear, but may be explained by the following pathways. First, depressive symptoms have known neuroendocrine (i.e., dysregulated function of the hypothalamic-pituitary-adrenocortical (HPA) axis) and inflammatory effects [42]. Studies suggested that depressive symptoms are positively associated with inflammatory factors (i.e., C-reactive protein, IL-6, and Tumor necrosis factor alpha) [42, 43], which are related to stroke [43]. In addition, depressive symptoms can dysregulate the HPA axis, thereby leading to abnormal changes in glucocorticoids and circulating catecholamine, which have an influence on endothelial function and platelet aggregation [44, 45]. Thus, patients with higher levels of depressive symptoms may experience higher risk of stroke. Second, depressed individuals typically have more health-related risk profiles, including smoking [10], drinking [46], physical inactivity [42], poor diet [42, 47], obesity [42], lower heart rate variability (HRV) [48], hypertension and other certain comorbidities [12, 49]. However, after adjusting for baseline characteristics of smoking, drinking frequency, BMI, hypertension, diabetes, and heart diseases, depressive symptoms’ effect on stroke incidence remained robust in our study, which indicated that the effect of depressive symptoms on stroke risk was independent of these variables. Finally, it is difficult for depressive patients to adhere to prescribed medical regimens (i.e., antihypertensive drugs) and depressive symptoms relate to individuals’ access to and quality of medical care [11, 50, 51], which might be another potential explanation for increased stroke incidents. Thus, future research to explore the specific mechanisms between depressive symptoms and stroke incidents are still necessary.

### Strengths and limitations

Regarding strengths, this was the first study to examine the associations between the changes in depressive symptoms and stroke risk in middle-aged and older populations in China. With community-based design and large sample, it allowed us to extrapolate our findings to the general population of this age. Moreover, by classifying depressive symptoms into different groups reflecting the change or stable aspects of depressive symptoms, and within the narrow time window, we were able to detect the effect of short-term depressive symptoms on stroke risk.

However, this study presents the following limitations: First, the stroke incident was defined by self-reported or proxy-reported doctor diagnosis, and it was impossible to confirm it via medical records or neuroimaging evidence, which might produce misclassification. Nevertheless, in a validation study of HRS, the bias of misclassification for self-reported stroke was moderate and acceptable [52]. In addition, previous studies reported a good validity of self-reported stroke by comparing self-reported stroke with stroke identified from multiple sources of information, including hospital records [53, 54]. Second, depressive symptoms were measured using the CESD-10 tool, which might lead to measurement errors in the ascertainment of depression. However, we used a higher cutoff (CESD-10 ≥ 12) for the definition of elevated depressive symptoms; the results were robust to sensitivity analyses. Furthermore, the prevalence of elevated depressive symptoms at baseline, in our study, is comparable to that expected for the population of this age [55]. Unfortunately, image data were not available in the CHARLS; thus, data for stroke types, such as hemorrhagic or ischemic stroke, were unavailable. Although this is common in most large-scale epidemiology studies, future research needs to determine the relationships between changes in depressive symptoms and stroke subtypes. Finally, the CHARLS included extensive data on demographic characteristics and potential stroke risk factors, allowing us to conduct analyses with multivariate adjustments; yet there may be some residential confounders we did not include, and exposures were self-reported, which may lead to some misclassification.

### Implications

Our findings that high depressive symptoms were associated with more stroke incidents than remitted depressive symptoms added new evidence to support prior studies that highlight the need for early intervention in middle-aged and older patients with depression. Stewart et al. reported that effective depression primary care may contribute to fewer cardiovascular disease events among older adults [56]. Unfortunately, due to multiple comorbidities [57], polypharmacy [58], and decreased social function [59], depression among older people is usually underdiagnosed and undertreated [60]. In addition, stable high and recent-onset depressive symptoms were present in 19.1 and 14.5% of participants in this study, respectively, and were associated with 2.01- and 1.39-fold increased stroke risk, respectively. The corresponding population-attributable risks due to exposure to stable high and recent-onset depressive symptoms were 16.2 and 5.4%, respectively. These estimates might provide an indication of the expected benefits of effective interventions for depressive symptoms in primary care. Nevertheless, few studies have examined the impact of interventions for depressive symptoms on stroke risk; further studies are needed to investigate this topic.

China is rapidly transforming into an aging nation, and there will be a surge in the prevalence and incidence of age-associated diseases, including chronic diseases and mental health disorders [61]. For example, chronic diseases lead to nearly 80% of total deaths and 70% of total disability-adjusted life years, with stroke being the leading cause of death in 2017 [5]. Urgent action is needed to develop a comprehensive program for the prevention of chronic diseases. In the meantime, Chinese households are experiencing rapid socioeconomic transition and the traditional family has been eroded in recent years. Thus, due to the lack of family support and poor health status, depressive symptoms are growing among older people [62]. Depression is more likely to be neglected than other aging diseases, in particular when individuals with depression are less likely to seek therapy due to the stigma of mental illness in some places in China [63]. Therefore, attention should be paid to mental disorders in Chinese older people, raising public awareness, launching public education programs, enhancing family and community support, and providing training for primary care staff to address mental health issues.

## Conclusions

In conclusion, our findings contribute to the limited evidence on the association between changes in depressive symptoms and stroke risk. We found that sustained high and recent-onset depressive symptoms were associated with increased stroke risk in adults aged 45 and older in China, while remitted depressive symptoms were not associated with higher risk, thereby suggesting that the risk of stroke may be decreased by effective management of depressive symptoms. Our results are also important to the scarce research on this association in China, where interventions and support to address mental health in primary care are required given the aging population and increasing burden of stroke.

## Supplementary Information

**Additional file 1 Supplementary file: Table I.** Baseline characteristics of sample population, stratified by elevated depressive symptoms of 2013, *n* = 10,100. **Table II.** Odds Ratios and 95%CI for associations between baseline depressive symptoms and stroke incidents. **Table III.** Subgroup analyses for association between baseline depressive symptoms and stroke risk. **Table IV.** Subgroup analysis for associations between different depressive symptom patterns and stroke risk. **Table V**. Sensitivity analyses with alternative cutoff of CESD-10 ≥ 12. **Table S1.** Baseline characteristics of participants before and after exclusion.

**Additional file 2 Appendix 1**: STROBE Statement —checklist of items that should be included in reports of observational studies.

## Data Availability

The data that support the findings of this study are available from the China Health and Retirement Longitudinal Study (CHARLS) group, but restrictions apply to the availability of these data, which were used under license for current study, and so are not publicly available. Data are however available from the authors upon reasonable request and with the permission of the CHARLS group.

## Abbreviations

CHARLS: Chinese Health and Retirement Longitudinal Study
HRS: Health and Retirement Study
CAPI: computer-assisted personal interviews
CESD-10: 10-item version of the Center for Epidemiological Studied Depression scale
BMI: body mass index
CDC: Chinese Center for Disease Control and Prevention
OR: odds ratio
SPSS: Statistical Package for the Social Sciences
MHAS: Mexican Health and Aging Study
CHS: Cardiovascular Health Study
