# Supplementary material for: Prospective association between depressive symptoms and stroke risk among middle-aged and older Chinese
Source: BMC Psychiatry. 2021 Oct 27;21:532. doi: 10.1186/s12888-021-03492-9 (PMC8555147; doi:10.1186/s12888-021-03492-9)
Supplement: Supplementary file 1 — Additional file 1 Supplementary file: Table I. Baseline characteristics of sample population, stratified by elevated depressive symptoms of 2013, n = 10,100. Table II. Odds Ratios and 95%CI for associations between baseline depressive symptoms and stroke incidents. Table III. Subgroup analyses for association between baseline depressive symptoms and stroke risk. Table IV. Subgroup analysis for associations between different depressive symptom patterns and stroke risk. Table V. Sensitivity analyses with alternative cutoff of CESD-10 ≥ 12. Table S1. Baseline characteristics of participants before and after exclusion. [file 12888_2021_3492_MOESM1_ESM.docx]

**Supplementary file**

**Table I. Baseline characteristics of sample population, stratified by elevated depressive symptoms of 2013, n=10,100**

| Characteristics | Overall, n=10,100 | Elevated depressive symptoms | | P value |
| --- | --- | --- | --- | --- |
|  |  | No, n=7001 | Yes, n=3099 |  |
| Age, Mean (SD) | 59.6(8.7) | 59.5(8.7) | 59.9(8.6) | .055 |
| Female, n (%) | 5425(53.7) | 3435(49.1) | 1990(64.2) | <.001 |
| Married, n (%) | 9025(89.4) | 6365(90.9) | 2660(85.8) | <.001 |
| Above high school education, n (%) | 1168(11.6) | 882(12.6) | 286(9.2) | <.001 |
| Live in rural, n (%) | 9311(92.2) | 6372(91.0) | 2939(94.8) | <.001 |
| Smoking status, n (%) |  |  |  | <.001 |
| Never smoked | 6150(59.3) | 4080(58.3) | 2070(66.8) |  |
| Current smoker | 3125(30.9) | 2298(32.8) | 827(26.7) |  |
| Past smoker | 825(8.2) | 623(8.9) | 202(6.5) |  |
| Drinking status, n (%) |  |  |  | <.001 |
| Never | 5989(59.3) | 4001(57.1) | 1988(64.1) |  |
| Rarely | 813(8.0) | 579(8.3) | 234(7.6) |  |
| Often | 3298(32.7) | 2421(34.6) | 877(28.3) |  |
| BMI, Mean (SD) | 24.0(4.7) | 24.1(4.9) | 23.7(4.1) | <.001 |
| Hypertension, n (%) | 4512(44.7) | 3141(44.9) | 1371(44.2) | .560 |
| Diabetes, n (%) | 1195(11.8) | 823(11.8) | 372(12.0) | .721 |
| Heart diseases, n (%) | 1264(12.5) | 778(11.1) | 486(15.7) | <.001 |

Abbreviations; SD=standard deviation.

Chi-square tests were used for categorical variables, and t -tests were for continuous characteristics.

**Table II.** **Odds Ratios and 95%CI for associations between baseline depressive symptoms and stroke incidents**

| **Baseline depressive symptoms** | **Model 1** | | **Model 2** | | **Model 3** | |
| --- | --- | --- | --- | --- | --- | --- |
|  | **OR (95%CI)** | **P value** | **OR (95%CI)** | **P value** | **OR (95%CI)** | **P value** |
| CESD-10<10 | Ref |  | Ref |  | Ref |  |
| CESD-10 ≥10 | 1.52(1.27,1.81) | <.001 | 1.52(1.27,1,82) | <.001 | 1.53(1.28,1.84) | <.001 |
| Continuous CESD-10 score | 1.04(1.02,1.05) | <.001 | 1.04(1.02,1.05) | <.001 | 1.04(1.02,1.05) | <.001 |

Abbreviations; OR=odds ratio, CI=confidence interval.

The model 1 was unadjusted. The model 2 was adjusted by baseline demographic variables including age, gender, education, marital status, place of residence. The model 3 was further adjusted by smoking status, drinking frequency, body mass index, hypertension, diabetes and heart disease.

**Table III. Subgroup analyses for association between baseline depressive symptoms and stroke risk**

|  | OR (95%CI) | P value | P for interaction |
| --- | --- | --- | --- |
| Age |  |  |  |
| 45-59 | 1.76(1.31,2.37) | <.001 | .950 |
| ≥ 60 | 1.39(1.10,1.75) | .006 |  |
| Gender |  |  | .414 |
| male | 1.41(1.06,1.88) | .018 |  |
| female | 1.62(1.28,2.07) | <.001 |  |
| Place of residence |  |  | .189 |
| rural | 1.47(1.22,1.78) | <.001 |  |
| urban | 2.72(1.37,5.39) | .004 |  |

Abbreviations; OR=odds ratio, CI=confidence interval.

The model was adjusted by baseline characteristics including age, gender, education, marital status, region, smoking status, drinking frequency, body mass index, hypertension, diabetes and heart disease.

**Table IV.** **Subgroup analysis for associations between different depressive symptom patterns and stroke risk**

|  | Stable low | Recent onset |  | Recently remitted |  | Stable high |  | P for interaction |
| --- | --- | --- | --- | --- | --- | --- | --- | --- |
|  | OR (95%CI) | OR (95%CI) | P value | OR (95%CI) | P value | OR (95%CI) | P value |  |
| Age |  |  |  |  |  |  |  | .220 |
| 45-59 | Ref | 1.32(0.82,2.12) | .247 | 0.81(0.44,1.49) | .498 | 2.26(1.55,3.31) | <.001 |  |
| ≥ 60 | Ref | 1.41(0.97,2.06) | .072 | 1.27(0.83,1.94) | .278 | 1.70(1.22,2.36) | .002 |  |
| Gender |  |  |  |  |  |  |  | .725 |
| male | Ref | 1.28(0.81,2.02) | .298 | 1.09(0.64,1.86) | .751 | 2.22(1.50,3.29) | <.001 |  |
| female | Ref | 1.44(0.98,2.13) | .063 | 1.05(0.66,1.67) | .840 | 1.84(1.33,2.53) | <.001 |  |
| Region |  |  |  |  |  |  |  | .103 |
| rural | Ref | 1.35(1.00,1.83) | .051 | 1.04(0.72,1.49) | .840 | 1.83(1.41,2.37) | <.001 |  |
| urban | Ref | 1.84(0.56,6.02) | .311 | 1.81(0.48,6.85) | .383 | 4.65(1.89,11.42) | .001 |  |

Abbreviations; OR=odds ratio, CI=confidence interval.

The stable low/no group was used as the reference. The model was adjusted by baseline characteristics including age, gender, education, marital status, region, smoking status, drinking frequency, body mass index, hypertension, diabetes and heart disease.

**Table V. Sensitivity analyses with alternative cutoff of CESD-10 ≥ 12**

|  | **Model 1** | | **Model 2** | | **Model 3** | |
| --- | --- | --- | --- | --- | --- | --- |
| Depressive symptom category | OR (95%CI) | P value | OR (95%CI) | P value | OR (95%CI) | P value |
| Stable low | Ref |  | Ref |  | Ref |  |
| Recent onset | 1.60(1.22, 2.10) | .001 | 1.60(1.21, 2.11) | .001 | 1.53(1.16, 2.03) | .003 |
| Recently remitted | 0.98(0.68, 1.41) | .915 | 0.98(0.68, 1.41) | .910 | 0.98(0.68, 1.42) | .919 |
| Stable high | 2.06(1.60, 2.65) | <.001 | 2.07(1.60, 2.69) | <.001 | 2.03(1.56, 2.64) | <.001 |
| Baseline elevated depressive symptoms |  |  |  |  |  |  |
| No | Ref |  | Ref |  | Ref |  |
| Yes | 1.36(1.12,1.65) | .002 | 1.35(1.11,1.65) | .003 | 1.36(1.12,1.66) | .002 |

Abbreviations; OR=odds ratio, CI=confidence interval, Ref: reference.

The model 1 was unadjusted. The model 2 was adjusted by baseline demographic variables including age, gender, education, marital status, place of residence. The model 3 was further adjusted by smoking status, drinking frequency, body mass index, hypertension, diabetes and heart disease.

**Table S1**. **Baseline characteristics of participants before and after exclusion.**

| Characteristics | All participants  (n=18,605) | Baseline depressive symptoms and stroke (n=10,100) | Changes of depressive symptoms  and stroke(n=8,491) |
| --- | --- | --- | --- |
|  |  |  |  |
| Age (mean) | 60.1 | 59.6 | 59.4 |
| Gender (%) |  |  |  |
| male | 47.7 | 46.3 | 44.8 |
| female | 52.3 | 53.7 | 55.2 |
| Place of residence (%) |  |  |  |
| rural | 89.8 | 92.2 | 92.3 |
| urban | 10.2 | 7.8 | 7.7 |
| Marital status (%) |  |  |  |
| married | 86.8 | 89.4 | 89.9 |
| single | 13.2 | 10.6 | 10.1 |
| Education (%) |  |  |  |
| below high school | 88.1 | 88.4 | 88.5 |
| high school and above | 11.9 | 11.6 | 11.5 |
